# Development and validation of the suicidal behaviours questionnaire - autism spectrum conditions in a community sample of autistic, possibly autistic and non-autistic adults

**DOI:** 10.1186/s13229-021-00449-3

**Published:** 2021-06-21

**Authors:** Sarah A. Cassidy, Louise Bradley, Heather Cogger-Ward, Jacqui Rodgers

**Affiliations:** 1 School of Psychology, University of Nottingham, University Park, Nottingham, NG7 2RD UK; 2 The International Centre, University of Bedfordshire, Luton, UK; 3 School of Psychology, University of Lincoln, Lincoln, UK; 4 Lincolnshire Partnership NHS Foundation Trust, Lincoln, UK; 5 Population Health Sciences Institute, Newcastle University, Newcastle upon Tyne, UK

**Keywords:** Suicidality, Autism spectrum conditions, Autistic traits, Measurement properties, Suicidal behaviours questionnaire, Measurement invariance

## Abstract

**Background:**

Autistic people and those with high autistic traits are at high risk of experiencing suicidality. Yet, there are no suicidality assessment tools developed or validated for these groups.

**Methods:**

A widely used and validated suicidality assessment tool developed for the general population (SBQ-R), was adapted using feedback from autistic adults, to create the Suicidal Behaviours Questionnaire—Autism Spectrum Conditions (SBQ-ASC). The adapted tool was refined through nine interviews, and an online survey with 251 autistic adults, to establish clarity and relevance of the items. Subsequently, 308 autistic, 113 possibly autistic, and 268 non-autistic adults completed the adapted tool online, alongside self-report measures of autistic traits (AQ), camouflaging autistic traits (CAT-Q), depression (PHQ-9), anxiety (ASA-A), thwarted belongingness and perceived burdensomeness (INQ-15), lifetime non-suicidal self-injury, and the original version of the suicidality assessment tool (SBQ-R). Analyses explored the appropriateness and measurement properties of the adapted tool between the groups.

**Results:**

There was evidence in support of content validity, structural validity, internal consistency, convergent and divergent validity, test–retest validity, sensitivity and specificity (for distinguishing those with or without lifetime experience of suicide attempt), and hypothesis testing of the adapted tool (SBQ-ASC) in each group. The structure of the SBQ-ASC was equivalent between autistic and possibly autistic adults, regardless of gender, or use of visual aids to help quantify abstract rating scales.

**Limitations:**

The samples involved in the development and validation of the adapted tool were largely female, and largely diagnosed as autistic in adulthood, which limits the generalisability of results to the wider autistic population. The SBQ-ASC has been developed for use in research and is not recommended to assess risk of future suicide attempts and/or self-harm. The SBQ-ASC has been designed with and for autistic and possibly autistic adults, and is not appropriate to compare to non-autistic adults given measurement differences between these groups.

**Conclusions:**

The SBQ-ASC is a brief self-report suicidality assessment tool, developed and validated with and for autistic adults, without co-occurring intellectual disability. The SBQ-ASC is appropriate for use in research to identify suicidal thoughts and behaviours in autistic and possibly autistic people, and model associations with risk and protective factors.

**Supplementary Information:**

The online version contains supplementary material available at 10.1186/s13229-021-00449-3.

## Introduction

People diagnosed with autism (henceforth autistic people^1^) are at significantly increased risk of experiencing suicidal thoughts and behaviours [3–5] and death by suicide [6–8], compared to non-autistic people. Autistic people who have experienced delay in autism diagnosis until adulthood show the highest estimates of lifetime suicidal thoughts (66%), and suicide attempt(s) (35–36%) [9, 10]. Many adults remain undiagnosed, given lack of adult autism diagnostic services and appropriate assessment tools to identify autism in women [11]. Possible undiagnosed autism is also associated with increased risk of suicidality. For example, 45% of women with high autistic traits in the region of clinical concern for possible autism reported making a suicide plan, and 16% had attempted suicide [12]. 40.6% of those with a lifetime history of suicide attempt(s), without autism diagnosis or suspected autism, scored above the clinical cut-off for possible autism on a validated measure of autistic traits [13]. 11% of depressed patients [14] and 15% of women with Borderline Personality Disorder (BPD) [15] met diagnostic criteria for co-occurring autism, and suicide attempts were highest in those with co-occurring autism diagnoses across both groups.

Given that autistic and possibly autistic people are at high risk of suicidality, it is crucial that appropriate and valid assessment tools are available to accurately identify suicidal thoughts and behaviours in these groups. However, systematic reviews have shown that no suicidality assessment tool has yet been validated for autistic people in research or clinical practice [16, 17]. Assessment tools are validated for certain purposes or circumstances, rather than being valid or invalid [16, 18]. Our aim was to develop a brief tool for use in research studies, rather than a brief risk assessment tool to predict future self-harm or suicide attempts in clinical practice. This decision was made because brief risk assessment tools are generally poor predictors of future self-harm or suicide attempts in clinical practice [19–21] and are therefore a “do not do” recommendation in current clinical guidelines in the UK [22]. Autistic people and those who support them also prioritised understanding risk and protective factors for suicidal thoughts and behaviours, how models of suicide apply to autistic people, and whether the experience of suicidality is different in autistic people [23, 24]. Addressing these community research priorities will require brief validated research tools. Additionally, answering these questions will provide crucial information for clinical practice, such as potentially unique risk factors for suicide in autistic people which need to be included in new effective risk assessment and treatment strategies [23, 24].

A recent systematic literature review [16] identified a suicidality assessment tool developed for non-autistic people—the Suicidal Behaviours Questionnaire—Revised [25]—as a promising candidate tool to adapt for autistic people for use in research studies. This was because: a) the SBQ-R was identified as the most frequently used tool in previous research, with a large body of evidence showing consistent moderate to strong evidence in support of a range of measurement properties when used in general population research, comparable to longer and more expensive tools; b) having a short research tool like the SBQ-R would reduce participant burden in research studies aiming to identify suicidal thoughts and behaviours; c) the SBQ-R was free to use and to adapt, crucial for facilitating future suicide in autism research; and d) some items of the SBQ-R in their current form had useful and specific definitions (e.g. “rarely (1 time)”, which previous research suggests could be particularly useful for autistic people [16]. The SBQ-R assesses lifetime experience of suicidal thoughts, plans and attempts (item 1), frequency of suicidal thoughts in the past year (item 2), communication of suicide intent to others (item 3), and likelihood of attempting suicide someday in the future (item 4).

One study has explored how autistic adults interpret and respond to the SBQ-R compared to non-autistic adults [26]. An online survey gathered responses to the SBQ-R in 188 autistic and 183 non-autistic adults matched on age and gender to compare the structure of the tool between the groups, and a subsample of 15 autistic adults were interviewed while completing the SBQ-R to explore how they interpreted and responded to the items. Results showed that the structure of the SBQ-R was significantly different in autistic compared to non-autistic adults, and autistic adults interpreted the items differently than intended by the scale developers. Specifically, autistic adults reported having difficulty communicating their suicide intent to others despite experiencing suicidality. Consistent with this, responses to item 3 (communication of suicidal intent) were less strongly associated with other items for autistic compared to non-autistic adults. This suggests that communication of suicidal intent to others is less strongly indicative of suicidality in autistic compared to non-autistic adults. Autistic adults said that it was important to ask about the likelihood of future suicide attempt(s) (item 4), but it was impossible to answer such an abstract future question. Likelihood of future suicide attempt(s) was more strongly associated with performance on other items in the autistic compared to the non-autistic group, which may indicate that autistic people are drawing more strongly on previous behaviour to inform their response. Autistic adults reported that item 2 did not capture the full range or intensity of suicidal thoughts over the past year, and difficulties with complex and imprecise response options across items, (e.g. did not want to/really hoped to die; never/no chance at all). Results also suggested a worrying clinical picture, that autistic adults reported impulsively attempting suicide without a plan when the means to attempt suicide where present [26].

Taken together these findings suggest that the SBQ-R would benefit from adaptation to improve the clarity and relevance of the items to autistic adults. The current study thus aimed to adapt the SBQ-R, incorporating feedback from autistic adults from the earlier study [26], and refining the adapted tool with additional interviews and an online survey. Subsequently, we assessed the appropriateness and measurement properties of the adapted Suicide Behaviours Questionnaire—Autism Spectrum Conditions (SBQ-ASC) in autistic adults in a new sample. A key issue in suicidality in autism research, is the lack of measurement tools available to assess and compare suicidality and associated risk markers between different groups. Given the high risk of suicidal behaviours in possibly autistic (but undiagnosed) adults, it is also important that the SBQ-ASC operates as intended in autistic people regardless of diagnosis. The current study therefore also assessed whether the structure of the adapted SBQ-ASC is measurement invariant (i.e. equivalent), and thus comparable between autistic, possibly autistic and non-autistic adults, and explored the measurement properties of the adapted tool in each group.

We hypothesised that autistic and possibly autistic adults would self-report significantly higher levels of suicidal thoughts and behaviours on the SBQ-ASC compared to non-autistic adults, on each item and total scores. Given the lack of previous suicidality research including possibly autistic people, we explored whether and how rates of suicidality differed between autistic and possibly autistic adults. We also hypothesised that total scores on the SBQ-ASC would be significantly correlated with risk markers for suicidality in autistic and non-autistic people identified from previous research (autistic traits, camouflaging autistic traits, depression, anxiety, non-suicidal self-injury (NSSI), thwarted belongingness and perceived burdensomeness) [27–35]. We also hypothesised that the SBQ-ASC would be more strongly correlated with the original version of the tool (given they both measure the same construct—suicidality), compared to other proximal risk markers for suicidality (e.g. thwarted belonging, perceived burdensomeness and mental health), which would in turn be more strongly correlated with the SBQ-ASC compared to more distal risk markers (e.g. autistic traits and camouflaging autistic traits). Given that we expected the SBQ-ASC to more accurately capture experience of suicidality relevant to autistic people, we also predicted that the SBQ-ASC would be more strongly correlated with autism relevant constructs (e.g. autistic traits, camouflaging autistic traits and measures developed for autistic people), compared to the original version of the tool. Lastly, given previous findings that autistic people might be more likely to impulsively attempt suicide without a plan, and have difficulties communicating their suicidal intent to others, we predicted that autistic and possibly autistic adults would be significantly more likely to endorse these sub-questions of the SBQ-ASC compared to non-autistic people.

## Method

### Ethical approval

The research received a favourable ethical opinion from the relevant local Research Ethics Committee (ethics approval references P47603 and F1074).

### Participants

The autistic group comprised 308 adults (27% male gender) who self-reported a diagnosis of ASC from a trained clinician. The possibly autistic group comprised 113 adults (29% male gender) who self-reported that they suspected they were autistic but had not yet been diagnosed. The non-autistic group comprised 268 adults (31% male gender) who reported that they were not diagnosed autistic or suspected they might be autistic. The autistic and possibly autistic groups were significantly more likely to identify with a different gender than assigned at birth (16.3%) compared to the non-autistic group (3%) (*X*^2^(1) = 28.79, *p* < 0.001; OR 6.24 CI 2.95–13.21). There was no significant difference in age between the three groups (*F*(688) = 1.34, *p* = 0.263). Self-reported autistic traits (AQ Scores) were significantly different between the three groups (*F*(682) = 601.59, *p* < 0.001). Bonferroni corrected *t *tests showed that the autistic group self-reported significantly higher autistic traits (mean = 22.86, SD = 3.76) compared to the possibly autistic (mean = 20.17, SD = 4.57) and non-autistic group (mean = 9.45, SD = 5.67), and the possibly autistic group self-reported significantly higher autistic traits than the non-autistic group (all *p* < 0.001) (Table 1).

**Table 1:** Participant characteristics

		Autistic	Possibly autistic	Combined autistic/possibly autistic	Non-autistic
		*N* = 308	*N* = 113	*N* = 421	*N* = 268
		N (%)/mean (SD)			
Questionnaires	AQ	22.86 (3.76)	20.17 (4.57)	22.14 (4.16)	9.45 (5.67)
	CAT-Q	129.02 (23.97)	123.5 (21.11)	127.56 (23.34)	83.31 (25.97)
	Thwarted belongingness	36.84 (10.65)	35.71 (10.81)	36.53 (10.69)	23.09 (11.26)
	Perceived burdensomeness	19.17 (9.21)	16.69 (8.51)	18.5 (9.09)	10.21 (5.68)
	ASA-A	37.82 (10.74)	32.49 (11.24)	36.4 (11.11)	19.35 (11.18)
	PHQ-9	14.29 (7.58)	12.92 (7.28)	13.92 (7.52)	7.37 (6.47)
Demographics	Sex	82 (26.62)	29 (25.66)	111 (26.37)	85 (31.72)
	Gender	83 (26.95)	33 (29.2)	116 (27.55)	83 (30.97)
	Identify with a different gender than birth sex	51 (16.56)	17 (15.04)	68 (16.15)	8 (2.99)
	Age (years)	39.71 (13.34)	40.34 (13.55)	39.88 (13.81)	41.57 (14.05)
	UK residency	243 (78.9)	73 (64.6)	316 (75.06)	182 (67.91)
Ethnicity	Asian	6 (1.95)	5 (4.42)	11 (2.61)	6 (2.24)
	Black or African or Caribbean	2 (0.65)	3 (2.65)	5 (1.19)	3 (1.12)
	Latinx	0 (0)	0 (0)	0 (0)	0 (0)
	Middle eastern or arab	2 (0.65)	0 (0)	2 (0.48)	2 (0.75)
	White or Caucasian	287 (93.18)	99 (87.61)	386 (91.69)	247 (92.16)
	Other ethnic group	12 (3.9)	5 (4.42)	17 (4.04)	10 (3.73)
	Prefer not to answer	2 (0.65)	2 (1.77)	4 (0.95)	1 (0.37)
Living arrangements	Living with flatmate(s)	9 (2.92)	9 (7.96)	18 (4.28)	15 (5.6)
	Living independently	88 (28.57)	23 (20.35)	111 (26.37)	50 (18.66)
	Living with a partner and/or dependent(s)	131 (42.53)	60 (53.1)	191 (45.37)	179 (66.79)
	Living with parents	55 (17.86)	11 (9.73)	66 (15.68)	20 (7.46)
	Living with friend(s)	7 (2.27)	5 (4.42)	12 (2.85)	2 (0.75)
	Living with a carer	1 (0.32)	1 (0.88)	2 (0.48)	0 (0)
	Living in supported accommodation	0 (0)	0 (0)	0 (0)	0 (0)
	Other	14 (4.55)	3 (2.65)	17 (4.04)	2 (0.75)
Employment status	Employed full time	93 (30.19)	47 (41.59)	140 (33.25)	154 (57.46)
	Employed part time	66 (21.43)	19 (16.81)	85 (20.19)	51 (19.03)
	Volunteering full time	1 (0.32)	1 (0.88)	2 (0.48)	1 (0.37)
	Volunteering part time	32 (10.39)	7 (6.19)	39 (9.26)	12 (4.48)
	Student full time	36 (11.69)	18 (15.93)	54 (12.83)	28 (10.45)
	Student part time	33 (10.71)	7 (6.19)	40 (9.5)	14 (5.22)
	Retired	14 (4.55)	6 (5.31)	20 (4.75)	21 (7.84)
	Unemployed looking for work	11 (3.57)	9 (7.96)	20 (4.75)	7 (2.61)
	Unemployed not looking for work	10 (3.25)	10 (8.85)	20 (4.75)	7 (2.61)
	Unable to work due to illness or disability	90 (29.22)	18 (15.93)	108 (25.65)	7 (2.61)
Education	Home	11 (3.57)	1 (0.88)	12 (2.85)	4 (1.49)
	Mainstream	296 (96.1)	107 (94.69)	403 (95.72)	259 (96.64)
	Special	12 (3.9)	1 (0.88)	13 (3.09)	2 (0.75)
	Other	8 (2.6)	6 (5.31)	14 (3.33)	7 (2.61)
	University degree	206 (66.88)	79 (69.91)	285 (67.7)	221 (82.46)
ASC subtype	Asperger syndrome	150 (48.7)			
	High functioning autism	29 (9.42)			
	Atypical autism	1 (0.32)			
	Autism	32 (10.39)			
	ASC	60 (19.48)			
	Classic autism	2 (0.65)			
	PDD-NOS	1 (0.32)			
	Other	30 (9.74)			
	Age of ASC diagnosis	34.58 (14.12)			
	Clinician confirmed	308 (100)			
Developmental conditions	> = 1 Co-occurring Developmental Condition	86 (27.92)	16 (14.16)	102 (24.23)	26 (9.7)
	Dyspraxia	24 (7.79)	4 (3.54)	28 (6.65)	5 (1.87)
	Learning disability	3 (0.97)	2 (1.77)	5 (1.19)	1 (0.37)
	Learning difficulty	1 (0.32)	0 (0)	1 (0.24)	0 (0)
	Dyscalculia	6 (1.95)	0 (0)	6 (1.43)	2 (0.75)
	Dyslexia	25 (8.12)	2 (1.77)	27 (6.41)	10 (3.73)
	ADHD	42 (13.64)	9 (7.96)	51 (12.11)	15 (5.6)
	Developmental delay	5 (1.62)	0 (0)	5 (1.19)	0 (0)
	Other	10 (3.25)	0 (0)	10 (2.38)	1 (0.37)
Current mental health/other conditions	> = 1 current mental health/other condition	220 (71.43)	61 (53.98)	281 (66.75)	86 (32.09)
	Depression	154 (50)	45 (39.82)	199 (47.27)	64 (23.88)
	Anxiety	184 (59.74)	50 (44.25)	234 (55.58)	57 (21.27)
	OCD	29 (9.42)	3 (2.65)	32 (7.6)	5 (1.87)
	Bipolar	14 (4.55)	3 (2.65)	17 (4.04)	3 (1.12)
	Personality disorder	20 (6.49)	9 (7.96)	29 (6.89)	8 (2.99)
	Schizophrenia	0 (0)	0 (0)	0 (0)	1 (0.37)
	PTSD	55 (17.86)	15 (13.27)	70 (16.63)	8 (2.99)
	Tourette's syndrome/tic disorder	6 (1.95)	0 (0)	6 (1.43)	1 (0.37)
	Anorexia	12 (3.9)	1 (0.88)	13 (3.09)	2 (0.75)
	Bulimia	5 (1.62)	1 (0.88)	6 (1.43)	3 (1.12)
	Other	30 (9.74)	5 (4.42)	35 (8.31)	4 (1.49)

AQ = Autism Spectrum Quotient; CAT-Q = Camouflaging Autistic Traits Questionnaire; TB = Thwarted Belonging; PB = Perceived Burdensomeness; ASA-A = Anxiety Scale for Autistic Adults; PHQ-9 = Patient Health Questionnaire—9 Items; ASC = Autism Spectrum Condition; PDD-NOS = Pervasive Developmental Disorder—not otherwise specified; ADHD = Attention Deficit and Hyperactivity Disorder; OCD = Obsessive Compulsive Disorder; PTSD = Post Traumatic Stress Disorder

The autistic and possibly autistic groups were recruited through the Cambridge Autism Research Database, the Autistica network, and social media. The non-autistic group was recruited through the Cambridge Psychology Research Database and social media channels.

### Measures

#### ***Suicide behaviours questionnaire***—***autism spectrum conditions***

**Fig. 1:**
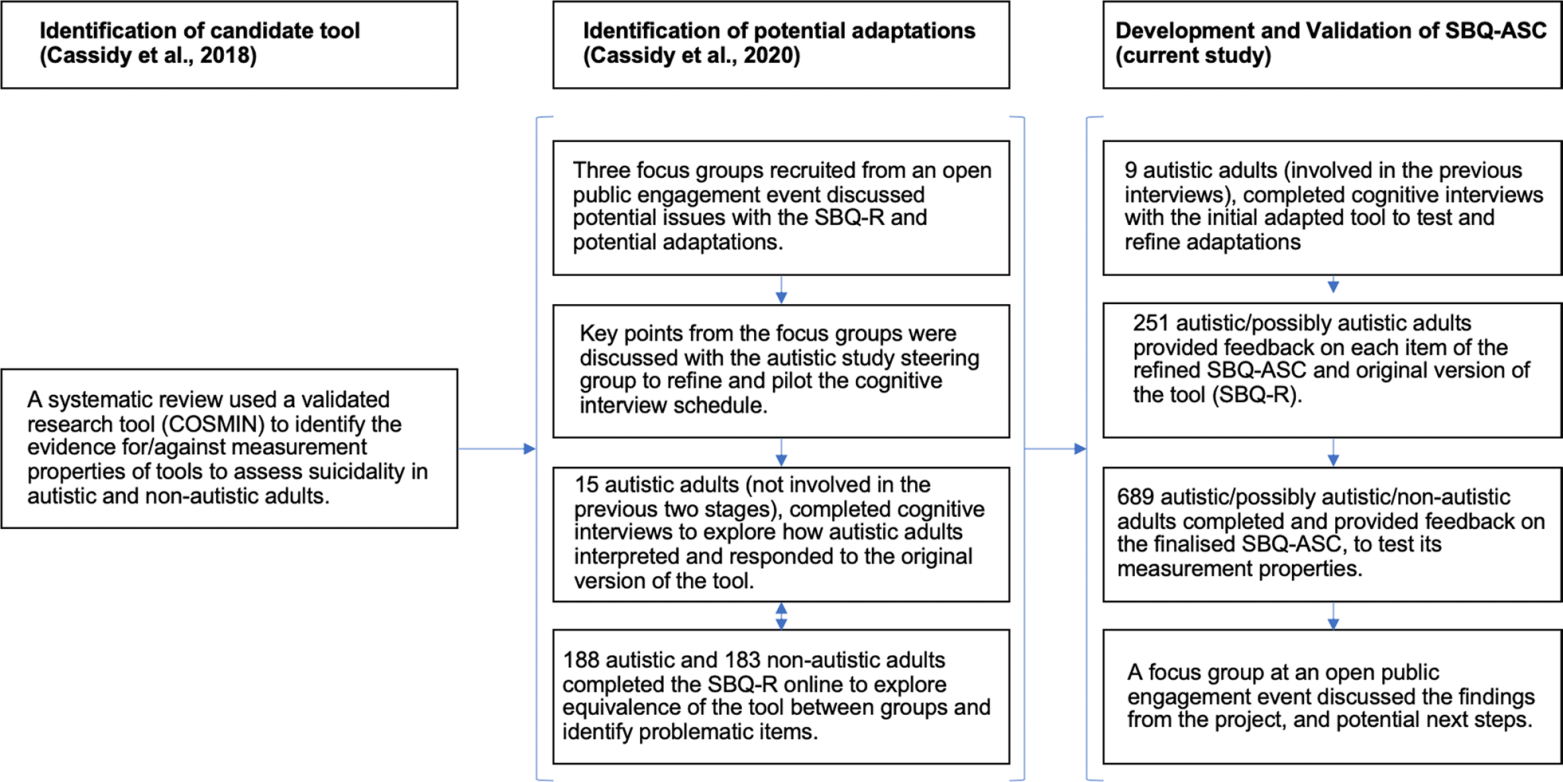
Stages of the overall research program to identify, develop and validate the SBQ-ASC from the original tool (SBQ-R)

Figure 1 shows the stages involved in the overall development and validation of the SBQ-ASC with and for autistic adults. The SBQ-ASC was adapted from the SBQ-R with permission of the tool developers [25]. A previous study had explored how autistic compared to non-autistic adults interpret and respond to the SBQ-R [26], to inform how to adapt this tool for autistic adults. The adapted SBQ-ASC was subsequently refined through: a) Cognitive interviews with 9 autistic adults (who took part in the earlier study, [26]); and b) A survey completed by 251 autistic and possibly autistic adults who provided qualitative feedback, and rated the clarity and importance of each item of the original SBQ-R and adapted SBQ-ASC (234 diagnosed, 17 awaiting assessment; 30.7% male; mean age = 41.91, *SD* = 13.44; mean age of ASC diagnosis = 36.09, *SD* = 14.03; 61.4% Asperger Syndrome diagnosis). Table 2 summarises the key issues identified with the SBQ-R by autistic adults across the interviews and online survey, and the subsequent adaptations incorporated into the SBQ-ASC to address these.

**Fig. 2:**
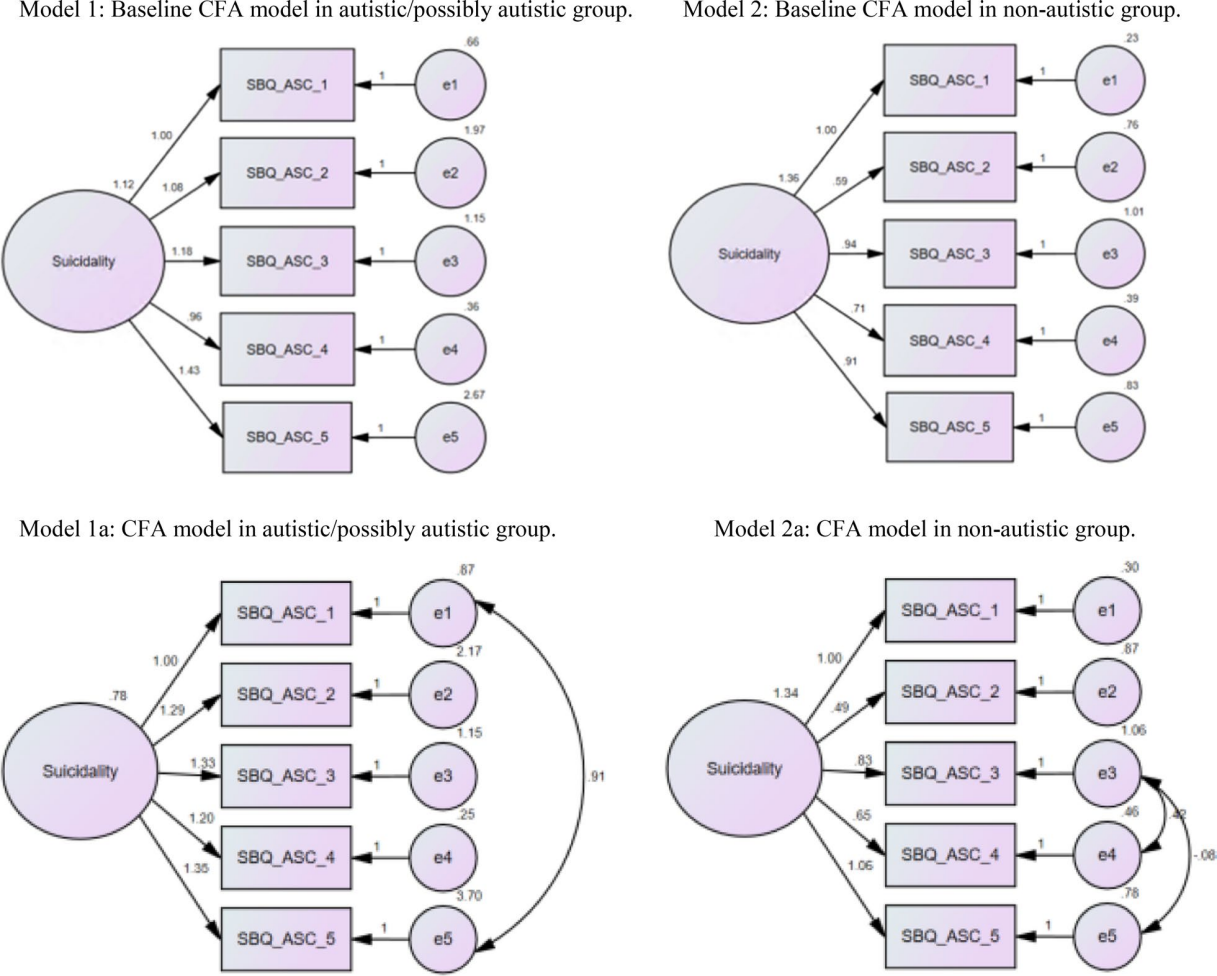
CFA models of the SBQ-ASC in the combined autistic/possibly autistic group, and the non-autistic group.

**Table 2:**
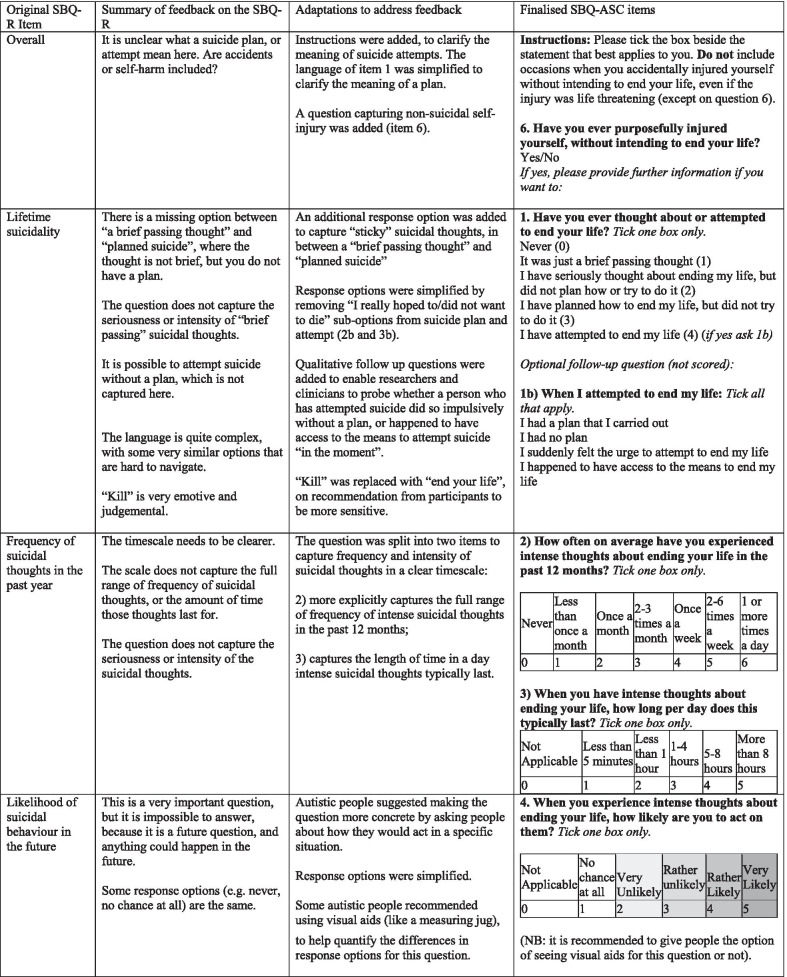

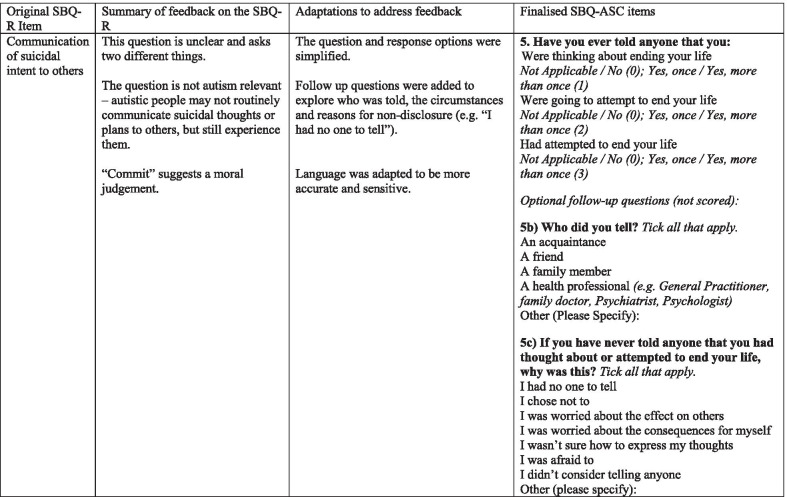
Summary of feedback across the cognitive interviews and survey on the SBQ-R and subsequent adaptations to the SBQ-ASC

The penultimate version of the SBQ-ASC items tested in the online survey was rated as “clear” by at least 79% of the 251 autistic/possibly autistic participants, and mean importance ratings ranged from 74.17 to 84.93 (out of 100) for each item (mean = 78.89, *SD* = 3.99). After minor corrections to grammar and wording to improve clarity, the final version of the SBQ-ASC used in the current study was given an overall mean clarity rating of 82.72 (out of 100, *SD* = 22.9) by the combined autistic/possibly autistic group, and 88.05 (*SD* = 21.13) by the non-autistic group. Analysis of the qualitative feedback across the two surveys showed that the issues identified with the penultimate version of the SBQ-ASC assessed in the earlier survey had been successfully addressed in the final version of the tool used in the current study.

The SBQ-ASC has five scored items (Table 2). Item 1 assesses lifetime experience of suicidal thoughts and behaviours from “Never” (0) to “I have attempted to end my life” (4). Item 2 assesses frequency of intense suicidal thoughts in the last 12 months from “Never” (0) to “1 or more times a day” (6). Item 3 assesses perseverative intense suicidal thoughts from “Not Applicable” (0), “Less than 5 min” (1) to “More than 8 h” (5). Item 4 assesses likelihood of suicide attempt from “Not Applicable” (0), “No chance at all” (1) to “Very likely” (5). It is recommended that a visual aid, such as a measuring jug or thermometer, is offered to participants to help quantify each response option for item 4 if they think this could be useful to them. Item 5 assesses communication of future suicide intent and past suicide attempts to others. Responses are scored from “Not applicable” (0)/“No” (0), to “Yes, once”/“Yes more than once”. Endorsing either “Yes” item is scored 1 for suicidal thoughts, 2 for future suicide attempts, and 3 for past suicide attempts. Participants can endorse all the options giving a maximum score of 6 for item 5.

Optional follow-up items which are not scored are also included in the SBQ-ASC. For those who endorse lifetime suicide attempt, these items address presence of plans, impulsivity and access to means. For those who have communicated suicidality to others, follow-up items gather information on who was told (e.g. friend, family member or professional). For those who have never told anyone about their suicidality, follow-up items gather information on why (e.g. I had no one to tell, I was afraid to). Item 6 also captures lifetime experience of non-suicidal self-injury, “Have you ever purposefully injured yourself, without intending to end your life? Yes/No. Therefore, alongside item 1, the SBQ-ASC can be used to classify lifetime experience of self-harm, with or without intent to end life.

#### ***Suicide behaviours questionnaire***—***revised (SBQ-R)***

The SBQ-R [25] is a four-item self-report questionnaire measuring suicidality. Item 1 assesses lifetime suicidal behaviour (on a scale from “Never” to “I have attempted to kill myself, and really hoped to die”). Item 2 assesses suicide ideation over the past 12 months (on a scale from “Never” to “Very Often (5 or more times)”). Item 3 assesses communication of suicidal intent to others (on a scale from “No” to “Yes, more than once, and really wanted to do it”). Item 4 assesses likelihood of a suicide attempt someday in the future (on a scale from “Never” to “Very likely”). The SBQ-R has been validated for use in general population samples to reliably distinguish people who have, from people who have not attempted suicide [25, 36]. The SBQ-R is widely used in research with moderate-strong evidence in support of internal consistency, structural validity, and criterion validity in research with general population samples [16]. The SBQ-R has also been utilised in research with autistic adults [e.g. 3,34], with evidence that the structure and interpretation of the SBQ-R is different in autistic compared to non-autistic adults [26]. Cronbach’s alpha for whole scale: Autistic group α = 0.739, possibly autistic group α = 0.755, non-autistic group α = 0.734.

#### ***Autism spectrum quotient***—***short (AQ-S)***

The AQ-short [37] is a 28-item subset of the full 50 item Autism Spectrum Quotient [38]. The AQ-28 measures the number of self-reported autistic traits, with high scores indicating more autistic traits. Items such as ‘it does not upset me if my daily routine is disturbed’ and ‘I find it easy to work out what someone is thinking or feeling’ are rated on a 4-item response scale from 1 “definitely agree” to 4 “definitely disagree” [37]. In the current study, responses endorsing autistic traits were given a score of 1, giving a total range from 0–28. A systematic review showed satisfactory evidence in support of the AQ-S factor structure, internal consistency, test–retest reliability and convergent validity as rated by a validated research tool (COSMIN) [39]. Using the dichotomous scoring method, scores at or above a clinical cut-off of 16 have showed acceptable sensitivity and specificity in distinguishing autistic from non-autistic adults [40]. Cronbach’s alpha for whole scale: Autistic group α = 0.765, possibly autistic group α = 0.791, non-autistic group α = 0.854.

#### Camouflaging autistic traits questionnaire (CAT-Q)

The Camouflaging Autistic Traits Questionnaire (CAT-Q) is a 25-item self-report questionnaire assessing the extent to which a person engages in social camouflaging behaviours, validated in autistic and non-autistic adults with equivalent factor structure between the groups [41]. The CAT-Q captures three domains of social camouflaging: (1) “compensation” (behaviours used to compensate for autism-related difficulties in social situations); (2) “masking” (behaviours used to hide autistic characteristics or present a non-autistic personality to others); and (3) “assimilation” (behaviours used to fit in better with others and not “stand out” from the crowd). Participants rate each of the 25 questions on a seven-point Likert scale between “Strongly Agree” to “Strongly Disagree”. Responses are scored between 1 and 7, with higher scores for items which endorse presence of social camouflaging behaviour. Cronbach’s alpha for whole scale: Autistic group α = 0.919, possibly autistic group α = 0.9, non-autistic group α = 0.931.

#### Anxiety scale for autism (Adults) (ASA-A)

The ASA-A [42] is a 20-item self-report measure of anxiety designed with and for autistic adults, adapted from the Anxiety Scale for Autism Spectrum Disorder (ASC-ASD). The ASA-A measures four components of anxiety: Social Phobia (e.g. ‘I worry what other people think of me’), Anxious Arousal (e.g. ‘All of a sudden I feel really scared’), and Uncertainty (e.g. ‘I am anxious about unfamiliar things, people or places’). Each item is rated on a scale from “Never” (0)—“Always” (3), with total scores ranging from 0–60. Scores at or above 28 indicate clinically significant levels of [42]. The ASA-A has strong evidence in support of its measurement properties (factor structure, internal consistency, test–retest reliability, convergent and divergent validity) in autistic adults [42]. Cronbach’s alpha for whole scale: Autistic group α = 0.921, possibly autistic group α = 0.926, non-autistic group α = 0.943.

#### ***Patient health questionnaire***—***9 Item (PHQ-9)***

The Patient Health Questionnaire-9 item (PHQ-9) [43] is a 9-item self-report scale used to assess severity of current depressive symptoms in line with DSM-V diagnostic criteria [44]. Scores range from 0 to 27 with scores at or over 10 indicating moderate, 15 moderately severe, and 20 severe depression. A recent systematic review showed that the PHQ-9 was extensively used in general population research, with strong evidence for its psychometric properties as rated by a validated research tool (COSMIN) [45], and more recently, evidence in support of total scores being comparable between autistic and non-autistic adults [46]. Cronbach’s alpha for whole scale: Autistic group α = 0.907, possibly autistic group α = 0.904, non-autistic group α = 0.909.

#### ***Interpersonal needs questions***—***15 item (INQ-15)***

The Interpersonal Needs Questionnaire (INQ-15) is a 15-item self-report questionnaire assessing ‘thwarted belongingness’ (e.g. ‘These days, I often feel like an outsider in social gatherings’) and ‘perceived burdensomeness’ (e.g. ‘These days, I think I am a burden on society’) [47]. The INQ-15 has been validated in young non-autistic adults [47] and has been used in previous research with autistic adults and those with high autistic traits [27, 33, 34, 48]. Cronbach’s alpha for whole scale: Autistic group α = 0.927, possibly autistic group α = 0.933, non-autistic group α = 0.936.

### Demographics

Participants were asked to report on their age, sex, gender, employment, education, living situation, diagnoses (developmental, mental health and other), ASC diagnosis (clinically confirmed, suspected but not yet unconfirmed, and not autistic or suspected to be autistic), and for those with clinically confirmed diagnosis, the age of ASC diagnosis and the type of professional they were diagnosed by (e.g. paediatrician, psychologist, psychiatrist).

### Procedure

Participants were invited to complete an online survey using Qualtrics aiming to adapt mental health assessment tools for autistic adults. Participants were informed that anyone 18-years or over could participate, regardless of autism diagnosis, experience of mental health problems or suicidal thoughts or behaviours. Participants were fully briefed about the nature of the research, that they could skip questions and sections of the survey that made them feel uncomfortable, stop the survey at any time and complete it later. Participants were also provided information about relevant support services before taking part in the study, after each section of the study, and after taking part in the study in a downloadable debrief sheet. After providing consent, participants completed the demographics questions, AQ-S, CAT-Q, INQ-15, ASA-A, PHQ-9, SBQ-R and SBQ-ASC. The order of the SBQ-R and SBQ-ASC were randomised between participants. Participants were then asked for consent to complete the SBQ-R and SBQ-ASC again in two weeks. Subsequently participants were provided with a full debrief including information about further information and support, followed by a positive mood induction procedure (a doodle page with jokes, puzzles and cute animal videos) which has proved effective in previous research exploring similar topics [49].

### Analyses

The autistic and non-autistic samples were split in two, stratified by gender and age. The first half was utilised in the exploratory factor analysis (‘exploratory sample’ *n* = 285, consisting of *n* = 153 autistic and *n* = 132 non-autistic adults), and the second half utilised in the confirmatory factor analysis (‘confirmatory sample’ *n* = 291, consisting of *n* = 155 autistic and *n* = 136 non-autistic adults). The two samples did not significantly differ in age (partial η^2^ = 0.001), autistic traits (partial η^2^ = 0.001), birth sex (OR = 0.84) or gender (Cramer’s V = 0.008). The confirmatory factor analysis model in the autistic group was subsequently tested in the whole possibly autistic group (*n* = 113). Analyses were conducted in SPSS version 26 and measurement invariance analysis conducted in SPSS AMOS version 24. 1319 participants initially accessed the survey. Of these, 748 participants who met eligibility criteria opted to see the SBQ-ASC questions, 689 (92.11%) of these participants completed all SBQ-ASC items with no missing data, and 686 also completed at least one additional measure with no missing items. Only measures with complete data for all items were included in the analysis. Prior to analysis, data for each measure were also screened for valid responses. This included checking that responses across questions were consistent (e.g. free text responses which addressed the question, that presence of lifetime suicidal thoughts were consistent across the SBQ-R and SBQ-ASC, and those who reported no lifetime suicidality also did not report suicidal thoughts in the past year etc.).

### Exploratory factor analysis of the SBQ-ASC

Principle components analyses were performed on the exploratory half of the autistic (*n* = 153), and non-autistic (*n* = 132) subsamples, and both the autistic and non-autistic groups combined (*n* = 285). The sample size was sufficient for EFA, with over 7 participants per item, and over 100 participants total [50]. Items with loadings below 0.4, or with cross-loadings of greater than 0.4 were excluded [51].

### Confirmatory factor analysis

The Chi-square statistic was used an indicator of fit [52], alongside other fit indices given that chi-square is affected by sample size [53]. The χ^2^*/df* ratio should be close to zero [54], root mean square of approximation (RMSEA) close to 0.06 [55], the comparative fit index higher than 0.9 [56], and Tucker-Lewis Index (TLI) values over 0.9 [52]. CFA was conducted on the confirmatory half of the autistic (*n* = 155), and non-autistic (*n* = 136) subsamples. The model identified in the autistic group was then tested in the whole possibly autistic group (*n* = 113). Groups that showed acceptable fit to the same model were combined, and the model re-run to test fit in the combined sample(s). Sample size was sufficient for CFA (> 7 participants per item, and > 100 participants total) [50].

### Measurement invariance

The exploratory and confirmatory samples were re-combined, and multi-group confirmatory factor analysis used to determine whether the SBQ-ASC had a similar structure between the groups: autistic (*n* = 308), possibly autistic (*n* = 113) and non-autistic (*n* = 268). Data was combined across groups which showed evidence for measurement invariance. Further analysis subsequently explored whether the structure was equivalent in males and females, and those who did and did not request visual aids for item 5 of the SBQ-ASC.

Measurement invariance analysis tests a series of nested models, with increasingly strict constraints, to assess evidence for increasingly strict levels of measurement invariance (i.e. equivalence) between groups [57, 58]: 1) *configural invariance* tests whether sets of items measure the same latent variable in both groups; 2) *metric invariance* tests whether the strength of the relationship between items are the same for both groups; 3) *scalar invariance* tests whether the total scores result from similar responses to individual items across groups; 4) *residual invariance* tests whether scale items measure the latent constructs with the same amount of measurement error across groups. In order to compare total scores from a measure between different groups, evidence for scalar invariance must be shown across the groups, as this suggests that total scores on the measure consists of similar performance on individual items [57]. Increase in RMSEA (> 0.015) and reduction in CFI (> 0.01) at each level were used as indicators of a significant degradation in fit, given that the chi-square statistic is strongly influenced by sample size [59].

### Reliability and validity

In each group, internal consistency was measured using Chronbach’s alpha for total scores. Spearman’s correlations, intraclass coefficient, and ANOVA assessed test–retest reliability of SBQ-ASC total scores between time one and time two in each group. Spearman’s correlations also assessed convergent validity between the SBQ-ASC with the original version of the tool (SBQ-R), and other measures of autistic traits (AQ), anxiety (ASA-A), depression (PHQ-9), lifetime NSSI (item 6 of the SBQ-ASC), thwarted belongingness and perceived burdensomeness (INQ-15). Divergent validity was assessed using z-tests to compare the strength of the correlation coefficients. Specifically: a) whether the SBQ-ASC was more strongly correlated with autism relevant constructs (AQ, CAT-Q and ASA-A) compared to the original version of the tool (SBQ-R); b) whether the correlation between the SBQ-ASC with the original version of the tool (SBQ-R) was larger compared to other proximal risk markers for suicide (thwarted belongingness, perceived burdensomeness, depression and anxiety); and c) whether the SBQ-ASC was more strongly correlated with more proximal mental health risk markers for suicide (depression and anxiety) compared to more distal risk markers (autistic traits and camouflaging autistic traits).

Receiver Operating Curve (ROC) analysis was used to establish an indicative cut-off score for the SBQ-ASC discriminating those who have from those who have not attempted suicide in their lifetime (using item 1 of the SBQ-R as the criterion). Kruskal Wallis analyses compared the SBQ-ASC items between groups, and total scores between groups (with evidence of measurement invariance at the scalar or residual level). Significant main effects were followed up with Mann Whitney U to test focused comparisons with partial eta squared calculated as a measure of effect size. Chi-square analyses compared frequency of sub-questions probing characteristics of suicidality, NSSI and above cut-off scores between the groups, with phi calculated as an estimate of effect size for multiple group comparisons, and odds ratios calculated as a measure of effect size for focused comparisons, with an alpha level of *p* < 0.01 to correct for multiple comparisons.

## Results

### Exploratory factor analysis

Table 3 shows the results of the EFA which indicates evidence for a one-factor solution (with all items loading above 0.4), explaining 57.86% of the variance in the autistic, 63.06% variance in the non-autistic group and 65.39% of the variance in both groups combined. All items of the SBQ-ASC were therefore retained.

**Table 3:** Item level factor loadings for the exploratory factor analysis in the autistic and non-autistic groups (exploratory sample)

SBQ-ASC item	Autistic group *N* = 153	Non-autistic group *N* = 132	Combined sample *N* = 285
	Factor loadings		
1. Have you ever thought about or attempted to end your life?	.817	.833	.854
2. How often on average have you experienced intense thoughts about ending your life in the past 12 months?	.692	.591	.715
3. When you have intense thoughts about ending your life, how long per day does this typically last?	.746	.869	.827
4. When you experience intense thoughts about ending your life, how likely are you to act on them?	.84	.878	.883
5. Have you ever told anyone that you: were thinking about ending your life/were going to attempt to end your life/had attempted to end your life?	.696	.764	.752
Cronbach’s Alpha	.818	.848	.85

### Confirmatory factor analysis

Examination of modification indices in the autistic and possibly autistic groups indicated that co-varying the error terms for conceptually related items 1 and 5 improved the fit of the model (1), whereas for the non-autistic group, co-varying error terms for items 3 and 5, and 4 and 5 improved the fit of the model (2) (Fig. 2). After co-varying the respective error terms, each group showed good fit to the model (Table 4).

**Table 4:** Model fit of confirmatory factor analysis in separate and combined groups (confirmatory sample)

Model	Model	*N*	*Χ*^*2*^	*df*	*Χ*^*2*^/*df* ratio	*p*	RMSEA	CFI	TLI
Autistic	0	155	35.92	5	7.18	.001	.2	.915	.829
Autistic	1	155	6.4	4	1.6	.171	.062	.993	.983
Possibly autistic	0	113	22.59	5	4.52	.001	.177	.924	.847
Possibly autistic	1	113	2.88	4	.721	.578	.001	1	1.01
Combined autistic/possibly autistic	0	268	52.46	5	10.49	.001	.189	.925	.85
Combined autistic/possibly autistic	1	268	4.36	4	1.09	.359	.018	.999	.999
Non-autistic	0	136	32.34	5	6.47	.001	.201	.927	.853
Non-autistic	1	136	18.32	4	4.58	.001	.163	962	.904
Non-autistic	2	136	2.87	3	.96	.412	.001	1	1.001

Recommended goodness of fit indices values demonstrating good model fit: χ^2^/df ratio close to zero (Bryant & Yarnold, 1995), RMSEA < 0.06, CFI > 0.95 and TLI > 0.9 (Browne, 2014; Hu & Bentler, 1993). Model 0 indicates baseline models without covaried error terms. Model 1 covaried error terms for items 1 + 5; Model 2 covaried error terms for items 3 + 4, and items 3 + 5 (Fig. 2)

### Measurement invariance analysis

Measurement invariance between the autistic and possibly autistic groups was tested, given the similar CFA model (1) identified in each of these groups separately and combined (Fig. 2 and Table 4). There was evidence for metric and scalar, but not residual invariance between the autistic and possibly autistic groups (Table 5). This suggests that the SBQ-ASC total scores can be compared between autistic and possibly autistic adults, and therefore data from these groups were combined in subsequent measurement invariance analyses. Measurement invariance analysis was not undertaken to compare the combined autistic/possibly autistic group to the non-autistic group, given the evidence for different baseline models in these groups (Fig. 2).

**Table 5:** Results of tests for measurement invariance in SBQ-ASC across the diagnosed autistic and possibly autistic adult groups

Model	*Χ*^*2*^	*df*	Model fit	CFI	TLI	ΔM	Model difference		*P*
			RMSEA				Δdf	Δ*χ*^*2*^	
M1: configural invariance (unconstrained)	7.56	8	.001	1	1.001				.478
M2: metric invariance	10.53	11	.001	1	1.001	M2–M1	3	2.97	.396
^1^M3: scalar invariance	11.67	12	.001	1	1.001	M3–M2	1	1.14	.286
M4: residual invariance	26.07	16	.039	.988	.985	M4–M3	4	14.41	.006

RMSEA = Root-mean-square error of approximation. CFI = comparative fit index. TLI = Tucker-Lewis Index

^1^Marginally significant degradation in fit is seen after this model (increase in RMSEA > .015 and reduction in CFI > .01)

Measurement invariance analysis therefore compared gender (males and females), and use of visual aids for item 4 of the SBQ-ASC, in the combined autistic/possibly autistic group, and separately in the non-autistic group. Analyses showed evidence for measurement invariance at the metric and scalar level when comparing gender in the combined autistic/possibly autistic group, and the non-autistic group, and evidence for measurement invariance at the metric, scalar and residual levels for the use of visual aids in the combined autistic/possibly autistic group, and the non-autistic group (Table 6).

**Table 6:** Results of tests for invariance in SBQ-ASC across gender and visual aids in the combined autistic/possibly autistic group, and non-autistic group

Model	*Χ*^*2*^	*df*	Model fit	CFI	TLI	ΔM	Model difference		*P*
			RMSEA				Δdf	Δ*χ*^*2*^	
*Comparison of gender (combined autistic/possibly autistic group)*									
M1: Configural invariance (unconstrained)	8.62	8	.015	.999	.998				.375
M2: Metric invariance	9.7	11	.001	1	1	M2–M1	3	1.08	.782
M3: Scalar invariance	11.03	12	.001	1	1	M3–M2	1	1.32	.25
M4: Residual invariance	12.02	16	.001	1	1.01	M4–M3	4	.999	.91
*Comparison of visual aids (combined autistic/possibly autistic group)*									
M1: Configural invariance (unconstrained)	5.58	8	.001	1	1.01				.694
M2: Weak factorial/metric invariance	7.31	11	.001	1	1.01	M2–M1	3	1.73	.631
M3: Scalar invariance	7.45	12	.001	1	1.01	M3–M2	1	.147	.7
M4: Strict invariance	13.73	16	.001	1	1	M4–M3	4	6.27	.18
*Comparison of gender (non-autistic group)*									
M1: Configural invariance (unconstrained)	19.38	6	.093	.98	.933				.004
M2: Weak factorial/metric invariance	23.68	9	.08	.978	.951	M2–M1	3	4.302	.231
^1^M3: Scalar invariance	24.28	10	.074	.979	.957	M3–M2	1	.595	.44
M4: Strict invariance	42.46	14	.089	.957	.939	M4–M3	4	18.18	.001
*Comparison of visual aids (non-autistic group)*									
M1: Configural invariance (unconstrained)	15.01	6	.075	.987	.955				
M2: Weak factorial/metric invariance	18.31	9	.062	.986	.969	M2–M1	3	3.297	.348
M3: Scalar invariance	18.41	10	.056	.987	.975	M3–M2	1	.105	.746
M4: Strict invariance	20.54	14	.042	.99	.986	M4–M3	4	2.132	.711

RMSEA = Root-mean-square error of approximation. CFI = comparative fit index. TLI = Tucker-Lewis index

^1^Marginally significant degradation in fit is seen after this model (increase in RMSEA > .015 and reduction in CFI > .01)

### Reliability and validity

Reliability and validity of the 5-item SBQ-ASC scale were explored in the combined sample of autistic/possibly autistic adults (*n* = 421), and non-autistic adults (*n* = 268), who had completed the SBQ-ASC and at least one other measure.

#### Internal consistency

Acceptable internal consistency was found for the total scale in the combined autistic/possibly autistic group (0.792) and the non-autistic group (0.848).

#### Test–retest reliability

All participants were invited to complete the SBQ-ASC two weeks after completing the first survey. Test–retest reliability was calculated in a sub-sample of autistic/possibly autistic participants (*n* = 172), and non-autistic participants (*n* = 72), who completed the SBQ-ASC 2-weeks after completing the initial survey. This sub-sample consisted of all participants who consented to complete the follow-up, and who completed all items of the SBQ-ASC at time one and time two. In the autistic/possibly autistic, and non-autistic groups, there were no significant differences in age, sex ratio, rate of any development or mental health condition, or questionnaire scores (AQ, CAT-Q, INQ-10, ASA-A, PHQ-9, SBQ-R) between participants who completed the SBQ-ASC at time one, compared to those who completed the SBQ-ASC at time one and time two (see Additional file 1 for results of all group comparisons).

Time one and time two SBQ-ASC scores were strongly correlated in the combined autistic/possibly autistic group (*r*_s_ = 0.927) and the non-autistic group (*r*_s_ = 0.902), with high intra-class correlations (autistic/possible autistic ICC = 0.928, 95% CI 0.9–0.946; non-autistic ICC = 0.921, 95% CI 0.877–0.95). There was no significant difference between SBQ-ASC total scores between time one and time two across both groups (*F*(242) = 1.34, *p* = 0.249), and no significant interaction between time points and group (*F*(242) = 2.3, *p* = 0.13).

#### Convergent validity

Spearman’s correlations were undertaken in the combined autistic/possibly autistic group, and the non-autistic group separately. SBQ-ASC total scores were significantly correlated with all measures in both groups and was highly correlated with the original version of the tool (SBQ-R) (Table 7).

**Table 7:** Inter-correlations between all variables in the combined autistic/possibly autistic group, and the non-autistic group

	AQ	CAT-Q	TB	PB	ASA-A	PHQ-9	SBQ-R	SBQ-ASC
*Autistic and possibly autistic group*								
CAT-Q	.233**							
TB	.172**	.067						
PB	.218**	.193**	.649**					
ASA-A	.282**	.453**	.279**	.485**				
PHQ-9	.181**	.209**	.568**	.670**	.585**			
SBQ-R	.141**	.197**	.441**	.634**	.393**	.573**		
SBQ-ASC	.164**	.232**	.384**	.631**	.460**	.545**	.877**	
NSSI	.122*	.232**	-.006	.237**	.228**	.172**	.286**	.319**
*Non-autistic group*								
CAT-Q	.659**							
TB	.515**	.505**						
PB	.393**	.445**	.737**					
ASA-A	.578**	.568**	.491**	.483**				
PHQ-9	.425**	.402**	.574**	.557**	.607**			
SBQ-R	.231**	.298**	.408**	.543**	.356**	.466**		
SBQ-ASC	.251**	.312**	.419**	.530**	.398**	.543**	.863**	
NSSI	.306**	.259**	.223**	.301**	.312**	.325**	.361**	.411**

AQ = autism spectrum quotient; CAT-Q = camouflaging autistic traits—questionnaire; TB = thwarted belongingness; PB = perceived burdensomeness; ASA-A = autism anxiety scale—adult; PHQ-9 = patient health questionnaire—9 item; SBQ-R = suicide behaviours questionnaire—revised; SBQ-ASC = suicide behaviours questionnaire—autism spectrum conditions; NSSI = non-suicidal self-injury

^**^p < .01; * *p* < .05

#### Divergent validity

In the autistic/possibly autistic group, the ASA-A was significantly more strongly correlated with the SBQ-ASC (*r*_s_ = 0.46), than the SBQ-R (*r*_s_ = 0.393) (*z* = 3.04, *p* < 0.001). There was no significant difference in the size of the correlation coefficient between the AQ/CAT-Q with the SBQ-ASC compared to the SBQ-R (AQ *r*_s_ = 0.164 vs. *r*_s_ = 0.141, *z* = 0.95, *p* = 0.171; CAT-Q *r*_s_ = 0.232 vs. *r*_s_ = 0.197, *z* = 1.465, *p* = 0.071 respectively).

The SBQ-ASC was significantly more strongly correlated with the SBQ-R (*r*_s_ = 0.877) than with thwarted belongingness (*r*_s_ = 0.384) (*z* = 13.77, *p* < 0.001), perceived burdensomeness (*r*_s_ = 0.631) (*z* = 10.76, *p* < 0.001), PHQ-9 (*r*_s_ = 0.545) (*z* = 11.984, *p* < 0.001) and ASA-A (*r*_s_ = 0.46) (*z* = 15.73, *p* < 0.001). The SBQ-ASC was significantly more strongly correlated with the PHQ-9 (*r*_s_ = 0.545) than with the AQ (*r*_s_ = 0.146) (*z* = 6.456, *p* < 0.001), and the CAT-Q (*r*_s_ = 0.232) (*z* = 6.257, *p* < 0.001). The SBQ-ASC was significantly more strongly correlated with ASA-A (*r*_s_ = 0.46) than with the AQ (*r*_s_ = 0.164) (*z* = 3.123, *p* < 0.001), but not the CAT-Q (*r*_s_ = 0.232) (*z* = 0.136, *p* = 0.446).

#### Sensitivity and specificity

ROC analysis showed that the SBQ-ASC had excellent sensitivity and specificity, with indicative cut-offs correctly classifying 88% of autistic adults who reported lifetime experience of suicide attempt(s) according to item 1 of the SBQ-R (Table 8).

**Table 8:** Results of the receiver operating curve (ROC) analysis

Group	SBQ-ASC cut-off	Sensitivity	Specificity	AUC	95% CI
Autistic/possibly autistic (* N* = 421)	11.5	0.867	0.74		
	**12.5**	**0.822**	**0.81**	.888	.855—.921
	13.5	0.77	0.85		

Bold indicates the recommended SBQ-ASC cutoff correctly classifying 88% of autistic adults who reported lifetime experience of suicide attempt(s) according to item 1 of the SBQ-R

#### Hypothesis testing

Table 9 includes response option endorsement, item means and mean total scores on the SBQ-ASC and results of all group comparisons between the autistic, possibly autistic and non-autistic groups. Autistic adults scored significantly higher than possibly autistic and non-autistic adults, and possibly autistic adults significantly higher than non-autistic adults, on items 1, 3, 4 and 5 of the SBQ-ASC (all *p* < 0.01). On item 2 of the SBQ-ASC, autistic and possibly autistic adults scored significantly higher than non-autistic adults (all *p* < 0.01). Autistic adults total scores on the SBQ-ASC were significantly higher than possibly autistic adults (*η*^*2*^ = 0.05). Autistic adults were also significantly more likely to score at or above the SBQ-ASC cut-off than possibly autistic adults (OR = 2.59) (*p* < 0.001).

**Table 9:** Comparison of individual item and total scores on the SBQ-R and SBQ-ASC between the autistic, possibly autistic and non-autistic groups

		Autistic	Possibly autistic	Combined autistic/possibly autistic	Non-autistic	Comparisons (autistic vs. possibly autistic vs. non-autistic)
		*N* = 308	*N* = 113	*N* = 421	*N* = 268	
		N (%)/Mean (SD)				
SBQ-R mean item scores	Lifetime suicidality	3.04 (.893)	2.66 (.935)	2.94 (.92)	2.11 (.965)	*H*(2) = 119.05, *p* < .001
	Frequency of suicidal thoughts in the past year	3.13 (1.59)^n^	2.78 (1.5)^n^	3.03 (1.57)	1.83 (1.21)	*H*(2) = 101.35, *p* < .001
	Suicide threat	1.78 (.865)	1.52 (.75)	1.71 (.841)	1.28 (.57)	*H*(2) = 52.9, *p* < .001
	Likelihood of future suicide attempt	2.85 (1.8)	2.08 (1.71)	2.65 (1.81)	1.06 (1.25)	*H*(3) = 146.65, *p* < .001
	Total SBQ-R score	10.81 (4.05)	9.04 (3.9)	10.33 (4.08)	6.28 (3.08)	-
	> Psychiatric cut-off	230 (76.7)	48 (57.1)	294 (71.4)	183 (30.9)	*X*^2^(2) = 119.27 *p* < .001, φ = .42
SBQ-ASC visual aids	Yes	117 (38)	35 (31)	152 (36.1)	59 (22)	*X*^2^(2) = 17.21 *p* < .001, φ = .16
SBQ-ASC item 1: lifetime suicidality	Non-suicidal	19 (6.17)	10 (8.85)	29 (6.89)	81 (30.22)	
	Suicidal ideation—"brief passing thought"	45 (14.61)	40 (35.4)	85 (20.19)	82 (30.6)	
	Suicidal ideation—"seriously thought about"	41 (13.31)	13 (11.5)	54 (12.83)	40 (14.93)	
	Suicide plan	90 (29.22)	24 (21.24)	114 (27.08)	42 (15.67)	
	Suicide attempt	113 (36.69)	26 (23.01)	139 (33.02)	23 (8.58)	
	Mean item score	2.76 (1.26)	2.14 (1.35)	2.59 (1.31)	1.42 (1.3)	*H*(2) = 125.98, *p* < .001
Lifetime suicide attempt subgroup follow-up questions:	I had a plan that I carried out	66 (58.41)	12 (46.15)	78 (56.12)	10 (43.48)	*X*^2^(2) = 2.74, *p* = .254, φ = .131
	I had no plan	19 (16.81)	1 (3.85)	20 (14.39)	3 (13.04)	*X*^2^(2) = 3, *p* = .223, φ = .136
	I suddenly felt the urge to attempt to end my life (impulsivity)	44 (38.94)	12 (46.15)	56 (40.29)	12 (52.17)	*X*^2^(2) = 1.49, *p* = .474, φ = .096
	I happened to have access to the means to end my life	31 (27.43)	9 (34.62)	40 (28.78)	5 (21.74)	*X*^2^(2) = 1.02, *p* = .6, φ = .08
SBQ-ASC item 2: frequency of suicidal thoughts in last 12 months	Never	90 (29.22)	39 (34.51)	129 (30.64)	173 (64.55)	
	Less than once a month	81 (26.3)	36 (31.86)	117 (27.79)	64 (23.88)	
	Once a month	29 (9.42)	16 (14.16)	45 (10.69)	13 (4.85)	
	2–3 times a month	32 (10.39)	8 (7.08)	40 (9.5)	8 (2.99)	
	Once a week	23 (7.47)	4 (3.54)	27 (6.41)	4 (1.49)	
	2–6 times a week	33 (10.71)	9 (7.96)	42 (9.98)	5 (1.87)	
	1 or more times a day	20 (6.49)	1 (0.88)	21 (4.99)	1 (0.37)	
	Mean item score	1.99 (1.96)^n^	1.41 (1.56)^n^	1.83 (1.88)	.06 (1.09)	*H*(2) = 100.25, *p* < .001
SBQ-ASC item 3: duration of suicidal thoughts	N/A	57 (18.51)	34 (30.09)	91 (21.62)	146 (54.48)	
	Less than 5 min	38 (12.34)	22 (19.47)	60 (14.25)	46 (17.16)	
	Less than 1 h	71 (23.05)	22 (19.47)	93 (22.09)	27 (10.07)	
	1–4 h	73 (23.7)	19 (16.81)	92 (21.85)	30 (11.19)	
	5–8 h	29 (9.42)	7 (6.19)	36 (8.55)	5 (1.87)	
	More than 8 h	40 (12.99)	9 (7.96)	49 (11.64)	14 (5.22)	
	Mean item score	2.32 (1.6)	1.73 (1.58)	2.16 (1.61)	1.04 (1.45)	*H*(2) = 95.7, *p* < .001
SBQ-ASC item 4: Likelihood of acting on suicidal thoughts	N/A	43 (13.96)	28 (24.78)	71 (16.86)	127 (47.39)	
	No chance at all	49 (15.91)	25 (22.12)	74 (17.58)	70 (26.12)	
	Very unlikely	95 (30.84)	42 (37.17)	137 (32.54)	45 (16.79)	
	Rather unlikely	90 (29.22)	17 (15.04)	107 (25.42)	25 (9.33)	
	Rather likely	27 (8.77)	1 (0.88)	28 (6.65)	1 (0.37)	
	Very likely	4 (1.3)	0 (0)	4 (0.95)	0 (0)	
	Mean item score	2.07 (1.21)	1.45 (1.05)	1.9 (1.2)	.89 (1.02)	*H*(2) = 128.38, *p* < .001
SBQ-ASC item 5: communication of suicidality to others	Suicidal ideation	186 (60.39)	50 (44.25)	236 (56.06)	82 (30.6)	
	Future suicide attempt	82 (26.62)	19 (16.81)	101 (23.99)	26 (9.7)	
	Past suicide attempt	110 (35.71)	24 (21.24)	134 (31.83)	26 (9.7)	
	Mean item score	2.21 (2.25)	1.42 (1.95)	1.99 (2.2)	.79 (1.47)	*H*(2) = 78.68, *p* < .001
	Overall communication of suicidality	210 (68.2)	57 (50.4)	267 (63.4)	92 (34.3)	
5b: Communicated suicidal intent subgroup: person(s) disclosed to	An acquaintance	5 (2.6)	4 (7.4)	9 (3.6)	3 (3.2)	*-*
	A friend	73 (37.2)	19 (35.2)	92 (36.8)	40 (42.6)	*X*^2^(2) = 1.03, *p* = .6, φ = .05
	A family member	75 (38.3)	17 (31.5)	92 (36.8)	37 (39.4)	*X*^2^(2) = 1.02, *p* = .6, φ = .05
	A health professional (e.g. General Practitioner, family doctor, Psychiatrist, Psychologist)	138 (70.4)	36 (63)	172 (68.8)	56 (62.3)	*X*^2^(2) = 3.65, *p* = .16, φ = .1
	Other	25 (12.8)	8 (14.8)	33 (13.2)	15 (16)	*X*^2^(2) = .582, *p* = .75, φ = .04
5c: Non-disclosure subgroup: reasons	I had no one to tell	22 (16.2)	4 (7.1)	26 (13.5)	6 (9)	*X*^2^(2) = 3.95, *p* = .14, φ = .12
	I chose not to	38 (27.9)	19 (33.9)	57 (29.7)	31 (46.3)	*X*^2^(2) = 6.72, *p* = .035, φ = .16
	I was worried about the effect on others	55 (40.4)	22 (39.3)	77 (40.1)	28 (41.8)	*X*^2^(2) = 081, *p* = .96, φ = .02
	I was worried about the consequences for myself	39 (28.7)	19 (33.9)	58 (30.2)	8 (11.9)	*X*^2^(2) = 9.3, *p* = .01, φ = .19
	I wasn't sure how to express my thoughts	39 (28.7)	17 (30.4)	56 (29.2)	7 (10.4)	*X*^2^(2) = 9.51, *p* = .009, φ = .19
	I was afraid to	27 (19.9)	8 (14.3)	35 (18.2)	5 (7.5)	*X*^2^(2) = 5.35, *p* = .07, φ = .14
	I didn't consider telling anyone	19 (14)	15 (26.8)	34 (17.7)	18 (26.9)	*X*^2^(2) = 6.65, *p* = .036, φ = .16
	Other (please specify):	17 (12.5)	7 (12.5)	24 (12.5)	4 (6)	*X*^2^(2) = 2.2, *p* = .33, φ = .09
SBQ-ASC item 6 (*n* = 602)	Lifetime NSSI	160 (62.7)	45 (46.4)	205 (58.2)	66 (26.4)	*X*^2^(2) = 67.46, *p* < .001, φ = .335
SBQ-ASC	Total score	11.34 (6.39)	8.15 (5.85)	10.5 (6.4)	4.75 (5.09)	*U* = 12,308.5, *p* < .001*
	> = SBQ-ASC autism cut-off	138 (44.8)	27 (23.9)	165 (39.2)	28 (10.4)	*X*^2^(2) = 85.02 *p* < .001, φ = .35

^n^Denotes non-significant focused comparisons (autistic vs. possibly autistic groups); ^*^Denotes focused comparison between autistic and possibly autistic total SBQ-ASC scores

Analysis of the optional sub-questions on the SBQ-ASC were compared between autistic, possibly autistic, and non-autistic adults. In the subsample who reported lifetime experience of suicide attempt(s) (*n* = 162), there were no significant between groups differences in the characteristics of past suicide attempt(s) (planning, impulsivity, or access to means) (all *p* > 0.22). In the sub-group who reported past communication of suicidal thoughts or behaviours to others (*n* = 344), there were no significant between group differences in who was disclosed to (acquaintance, friend, family member, professional or other) (all *p* > 0.21). In the sub-group who reported not having disclosed suicidal thoughts or behaviours to others (*n* = 259), compared to non-autistic adults, autistic and possibly autistic adults were significantly more likely to endorse “I was worried about the consequences for myself” (autistic vs. non-autistic OR = 2.96; possibly autistic vs. non-autistic OR = 3.79) and “I wasn’t sure how to express my thoughts” (autistic vs. non-autistic OR = 3.45; possibly autistic vs. non-autistic OR = 3.74), as reasons for non-disclosure (all *p* < 0.01). In the subgroup who reported lifetime history of NSSI (*n* = 602), autistic adults were significantly more likely to endorse lifetime experience of NSSI compared to possibly autistic (OR = 1.95) and non-autistic adults (OR = 4.69), and possibly autistic adults compared to non-autistic adults (OR = 2.41) (all *p* < 0.01).

## Discussion

To our knowledge, no suicidality assessment tool has previously been developed and validated with and for autistic adults for use in research, despite this group being at high risk of experiencing suicidal thoughts and behaviours [16]. A previous study showed that a widely used suicidality assessment tool developed and validated for the general non-autistic population for use in research studies (the SBQ-R), was not interpreted and responded to in the same way by autistic adults, and did not include items relevant to autistic adults’ experience of suicidality (e.g. perseverative suicidal thoughts, impulsive suicide attempts without a plan, why suicidality had not been disclosed to others) [26]. We therefore adapted the SBQ-R with and for autistic adults, and subsequently tested the measurement properties of the adapted SBQ-ASC, in autistic, possibly autistic and non-autistic adults. Our results show support for a range of measurement properties of the SBQ-ASC for use in research studies with autistic/possibly autistic adults. The SBQ-ASC can be freely downloaded from: https://sites.google.com/view/mentalhealthinautism/resources/tools.

Results show support for content validity of the SBQ-ASC in autistic/possibly autistic adults, with high ratings in support of the clarity of the adapted items (> 0.8). Cognitive interviews confirmed that autistic adults interpreted and responded to the adapted items as intended. Results subsequently showed support for the structural validity and internal consistency of the SBQ-ASC in autistic, possibly autistic and non-autistic adults. Exploratory and confirmatory factor analyses (in independent samples) showed excellent fit to a single factor structure for the SBQ-ASC in autistic, possibly autistic, and non-autistic adults, with acceptable internal consistency (> 0.79) in each group. There was evidence of a different baseline model in the combined autistic/possibly autistic group compared to the non-autistic group, indicating that the structure of the SBQ-ASC is different in these groups. There was evidence in support of measurement invariance (i.e. equivalence) of the SBQ-ASC in autistic compared to possibly autistic adults, males compared to females, and use of visual aids to help quantify response options for item 4 (e.g. no chance at all, and rather likely), in autistic/possibly autistic and non-autistic adults. This indicates that SBQ-ASC total scores can be compared and/or combined between autistic people with or without a confirmed diagnosis, across genders, and use of visual aids for item 4. However, total scores on the SBQ-ASC cannot be compared between autistic and non-autistic adults, given evidence for a different baseline model between these groups. The SBQ-ASC also showed excellent stability of scores, with strong correlations (> 0.9) between SBQ-ASC total scores pre/post a 2-week gap in autistic/possible autistic, and non-autistic adults.

There was evidence in support of convergent validity, with the SBQ-ASC significantly correlating with known risk markers for suicidality (autistic traits, camouflaging, thwarted belongingness and perceived burdensomeness, current anxiety and depressive symptoms, and lifetime NSSI), in both autistic/possibly autistic adults, and non-autistic adults. There was also evidence in support of divergent validity. Specifically, the ASA-A, an anxiety measure designed to more accurately identify anxiety in autistic adults [42], was significantly more strongly correlated with the SBQ-ASC (a measure also designed with and for autistic adults), compared to the SBQ-R (a measure designed for non-autistic adults). Autistic traits (AQ) and camouflaging autistic traits (CAT-Q), were also both more strongly correlated with the SBQ-ASC than with the SBQ-R, but these differences were not statistically significant. This suggests that the SBQ-ASC is more sensitive to detecting associations with autism relevant constructs compared to the original version of the tool, and is therefore more appropriate for use in suicidality research in autistic/possibly autistic samples than the original version.

There was further evidence of divergent validity, indicating that the SBQ-ASC is also sensitive to detecting differences in the strength of associations between proximal compared to more distally related constructs. The SBQ-ASC was significantly more strongly correlated with the original version measuring the same construct of suicidality (SBQ-R), compared to more proximal risk markers for suicidality (thwarted belongingness, perceived burdensomeness, depression and anxiety). The SBQ-ASC was also significantly more strongly correlated with more proximal risk markers for suicidality (depression/anxiety) than more distal risk markers (autistic traits). These results suggest that the SBQ-ASC could be particularly useful in modelling studies aiming to identify and distinguish proximal/distal risk markers for suicidal thoughts and behaviours in autistic/possibly autistic people—a crucial and underexplored area of research prioritised by the autism community [23, 24].

An indicative cut-off on the SBQ-ASC for distinguishing autistic/possibly autistic adults, with or without a lifetime history of suicide attempt(s), is 12.5. This cut-off showed excellent sensitivity and specificity, correctly classifying 88% of autistic/possibly autistic adults who self-reported lifetime experience of suicide attempt(s) using item 1 of the original SBQ-R as the criterion. This follows the recommendation of COSMIN (a validated research tool used to assess the methodological quality of studies exploring evidence for and against the measurement properties of health outcome assessment tools), to use the original version of an assessment tool as the ‘gold standard’ criterion on which to assess sensitivity and specificity [50]. This cut-off is appropriate to use in research studies to categorise autistic/possibly autistic adults in a sample at a higher/lower risk of lifetime suicide attempt(s), to help establish prevalence, and compare subgroups within the wider sample. However, this cut-off is *not* appropriate to be used in the context of treatment decisions or classifying autistic/possibly autistic people as high or low risk of future suicide attempts in clinical practice. The reasons being that first, this cut-off has been calculated in the context of research and past (not future) suicide attempt(s), and second, there is strong evidence that short suicide risk assessment tools like the SBQ-ASC and SBQ-R do not help clinicians correctly identify who will likely attempt suicide in the future [19–21].

The SBQ-ASC showed evidence in support of hypothesis testing. As predicted, autistic/possibly autistic adults scored significantly higher on each item of the SBQ-ASC than non-autistic adults. Interestingly, total scores on the SBQ-ASC were significantly higher in autistic compared to possibly autistic adults. The SBQ-ASC also shows strong potential for increasing our understanding of how suicidal thoughts and behaviours present in autistic/possibly autistic adults. Across the interviews and online surveys, autistic adults reported presence of perseverative suicidal thoughts, and impulsive suicide attempts without necessarily having a plan when the means to self-harm were present [26]. Autistic people also reported that they found it difficult to disclose their suicidal thoughts and behaviours to others, because of communication difficulties, social isolation, and lack of access to support. The SBQ-ASC includes optional items to explore and compare these experiences between groups. Contrary to our predictions, in the subgroup who reported lifetime experience of suicide attempts, there were no significant differences between autistic, possibly autistic and non-autistic adults in having a suicide plan, impulsivity or access to means. In the subgroup who had communicated suicidality to others, there were no significant differences between the groups in who was told (e.g. acquaintance, friend, family, professional). However, this may have been because of the lifetime focus of these questions, and possible measurement differences between items. Future research could ask these questions about specific instances of contemplating and/or attempting suicide, to compare the characteristics and patterns of suicidal thoughts and behaviours between autistic and non-autistic people.

Importantly, and in line with our hypotheses, in the subgroup who had never disclosed suicidality to others, autistic and possibly autistic adults were significantly more likely to report being worried about the consequences for themselves, and not being sure how to express their thoughts compared to non-autistic people. Previous research shows that autistic people experience high anxiety, and part of the reason is intolerance of uncertainty in the future [60]. In our interviews with autistic people, many reported anxiety about the purposes of assessments for suicidal thoughts and behaviours, and what would happen next. Communication difficulties are required for a diagnosis of autism [44], and autistic people can also experience difficulties verbalising their own internal thoughts and feelings (termed Alexythymia) [61]. Many autistic people in our interviews also described finding it difficult to communicate their suicidality to others, but nevertheless experiencing suicidality. Findings from the current study are consistent with these experiences, and show evidence of possibly different reasons for non-disclosure in autistic compared to non-autistic people. Importantly, the top reason for non-disclosure across all groups was worry about the impact of disclosing suicidality on others. Recent research has challenged assumptions of lack of empathy in autistic people [62, 63], and clinicians should not assume that this is not a similar obstacle to disclosure in autistic people.

There are clear implications for research and clinical practice. The SBQ-ASC is the first suicidality assessment tool developed and validated with and for autistic/possibly autistic adults for use in research. We would caution against using the SBQ-ASC alone to inform treatment decisions or to assess risk of future suicide attempts (see above) [22]. However, the SBQ-ASC could be helpful to clinicians in starting to identify presence of lifetime suicidal thoughts and behaviours, and suicidal thoughts in the past year, in autistic/possibly autistic adults. The optional follow-up questions included in the SBQ-ASC could also help clinicians gain useful initial information about previous suicide attempts (planning, impulsivity and access to means), whether and who the person has ever told about their suicidality, and reasons for non-disclosure, to start important conversations about possible support and safety planning (e.g. ways of alerting key people to suicidal crises, facilitating access to relevant support and social networks, addressing concerns and answering questions about what would happen in the event of reporting suicidal thoughts and/or behaviours). Clinicians should be aware of the different experience of suicidality in autistic people, who appear more likely to experience perseverative suicidal thoughts than non-autistic people. Broadly, clinicians should be aware that autistic/possibly autistic people are potentially more likely to be concerned about what will happen to them if they disclose their suicidality to others, have significantly more difficulty in knowing how to communicate their suicidal thoughts and intent to another person, and be concerned about the potential impact of disclosure on others. These likely present barriers to help seeking, and clinicians should therefore take the initiative and ask autistic people and those with high autistic traits (but not diagnosed) if they are experiencing suicidality.

A key strength of this study and broader program of work developing the SBQ-ASC, is the involvement of autistic people in first identifying the need for the research [23, 24], identifying potential issues with the original version [26], and co-producing adaptations in the current study. Assessing the appropriateness and measurement properties of the SBQ-ASC followed recommended best practice according to a validated research tool used to assess the quality of evidence for and against the measurement properties of health outcome assessment tools (COSMIN) [50]. COSMIN argues that content validity is the most important foundational property on which all other measurement properties rely on—if a tool is not relevant or clear to the target group, then it is unlikely to adequately capture the intended construct in that group. Despite this, few studies conduct extensive work to establish content validity of tools in groups, using rigorous methods such as participatory approaches [26, 64, 65] or cognitive interviewing [66]. In contrast, we conducted extensive work to ensure that the adapted SBQ-ASC captured the unique experience of suicidality in autistic and possibly autistic adults, prior to finalising the tool and assessing its measurement properties in autistic, possibly autistic and non-autistic adults. We also followed other key recommendations, such as using separate samples to explore and confirm the factor structure of the tool between groups, with at least 7 participants per item and over 100 participants total, and used the original version of the tool as the ‘gold standard’ criterion to assess sensitivity and specificity [50]. Results from the current study suggest that ensuring content validity increases the sensitivity of the new tool to detecting associations with relevant constructs (i.e. autistic traits).

In research, tools tend to be validated in one group, but used in many others, without necessarily testing whether the tool operates similarly between the groups being studied. Another key strength of the current study was testing whether the SBQ-ASC operates similarly between autistic/possibly autistic, and non-autistic adults. Results suggest important measurement differences between autistic/possibly autistic adults, compared to non-autistic adults. This is consistent with previous research showing that autistic adults do not interpret or respond as intended by the tool developers to the original version of the SBQ-R [26], or tools developed for non-autistic people more generally [65]. Key adaptations to the SBQ-ASC, informed from our previous research [26] and current study, included simplifying and clarifying response options, including visual aids to help quantify abstract response options (e.g. rarely, very likely), and including new items to capture the full range of frequency and duration of suicidal thoughts (up to every day, and over 8 h duration) reported by autistic adults. These adaptations follow best practice guidelines for adapting scales with and for autistic people [65], with new items developed to better capture autistic adults’ unique experiences of suicidality, not included in any previous measures designed for other groups. Results from the measurement invariance analysis therefore that suggest autistic people experience suicidality differently to non-autistic people, and this experience is not accurately captured in previous measures designed for non-autistic people. Future research must further explore autistic people’s unique experiences of constructs such as suicidality and mental health, to inform a broader suite of tools which better capture these constructs in different contexts, including clinical practice.

## Limitations

The study sample and wider program of work developing the SBQ-ASC included a high proportion of females, who were largely diagnosed in adulthood or were awaiting assessment. Participation involved interviews and completing online surveys. Therefore, results are likely not generalisable to autistic adults diagnosed in childhood, or with co-occurring intellectual disability. However, there is evidence that autistic women are at significantly increased risk of death by suicide compared to non-autistic women [6–8], and are more likely to experience delay in diagnosis due to lack of appropriate autism assessment tools validated for females [11]. Given the focus of the current study on developing an appropriate tool to effectively identify suicidal thoughts and behaviours in autistic/possibly autistic adults in research studies, it was crucial to include a large sample of women, which have been traditionally under-represented in autism research [67]. There were also sufficient numbers of males and females in the current study to establish equivalence of the adapted SBQ-ASC across gender according to best practice guidelines for the analyses [50]. Hence, the SBQ-ASC has been validated for both autistic and possibly autistic males and females. Adults without co-occurring intellectual disability, and/or diagnosed in adulthood, are at particularly high risk of suicidal thoughts and behaviours [3, 9] and death by suicide [6]. This suggests that the SBQ-ASC is particularly appropriate for identification and modelling of risk markers for suicidality in this group at relatively high risk of suicidality. However, future research will need to explore whether the SBQ-ASC is also appropriate for autistic people diagnosed in childhood, and/or co-occurring intellectual disability. Autism diagnostic status was confirmed through self-report. The possibly autistic group also consisted of those who self-identified as autistic, without a diagnosis. Future research could also include possibly autistic people who are suspected to be autistic by others (e.g. by family and friends).

The SBQ-ASC has been developed and validated for use in *research*. The SBQ-ASC has not been validated and there is no evidence in support of predicting future adverse events including suicide attempts using scores from this tool. The SBQ-ASC assesses lifetime experience of suicidal thoughts and behaviours, frequency of suicidal thoughts in the past year, duration of suicidal thoughts, likelihood of future suicide attempt, and communication of suicidal thoughts and behaviours to others. The SBQ-ASC does not however capture current suicidal thoughts. Further work will therefore be necessary to develop a range of suicidality assessment tools appropriate for different subgroups and contexts, including autistic children and youth, with or without intellectual disability, in research and clinical practice.

## Conclusion

We present a new tool developed and validated with and for autistic and possibly autistic adults, to more accurately capture suicidality in these groups in research studies—the SBQ-ASC. The SBQ-ASC was adapted from a well validated and widely used suicidality assessment tool originally developed for the general population for use in research studies (the SBQ-R) [25, 26]. The SBQ-ASC has evidence in support of a range of measurement properties, including content validity, structural validity, internal consistency, test–retest validity, convergent and divergent validity, criterion validity, and hypothesis testing. There is also evidence in support of the structural equivalence of the SBQ-ASC in autistic compared to possibly autistic adults, regardless of gender, and use of visual aids to assist with quantification of abstract response options for item 4 (e.g. likely vs. very likely). The SBQ-ASC is therefore recommended to identify suicidal thoughts, behaviours and characteristics in autistic adults (diagnosed or undiagnosed), without co-occurring intellectual disability, in research to help model risk factors for suicidality and associated characteristics. The SBQ-ASC could also be useful in clinical practice to help identify lifetime suicidal thoughts and behaviours, and past year suicidal thoughts, characteristics (plans, impulsivity, access to means, reasons for non-disclosure), and to start conversations about needed support (e.g. with removing access to means, and with help seeking in a crisis). However, results also suggest that the SBQ-ASC is not appropriate for comparing total scores between autistic/possibly autistic adults, with non-autistic adults given measurement differences between these groups. These findings suggest that autistic people experience suicidality differently to non-autistic people, and future research must further develop tools with and for autistic/possibly autistic adults, to better understand and capture unique experiences of suicidality and associated risk markers.

## Supplementary Information

**Additional file 1.** Results of group comparisons between follow-up survey responders and non-responders.

## Data Availability

The dataset used and analysed in the current study is available from the corresponding author on reasonable request.

## Abbreviations

ADHD: Attention deficit and hyperactivity disorder
AQ: Autism spectrum quotient
ASA-A: Anxiety scale for autism – adult
ASC: Autism spectrum condition
CAT-Q: Camouflaging autistic traits questionnaire
CFA: Confirmatory factor analysis
COSMIN: Consensus‐based standards for the selection of health measurement instruments
EFA: Exploratory factor analysis
INQ-15: Interpersonal needs questionnaire – 15 item
NSSI: Non-suicidal self-injury
OCD: Obsessive compulsive disorder
PTSD: Post traumatic stress disorder
PDD-NOS: Pervasive developmental disorder—not otherwise specified
PHQ-9: Patient health questionnaire – 9 item
PB: Perceived burdensomeness
ROC: Receiver operating curve
TB: Thwarted belongingness
SBQ-ASC: Suicidal behaviours questionnaire – autism spectrum conditions
SBQ-R: Suicide behaviours questionnaire – revised

## Footnotes

1 We use identity first language (autistic community/people/person) to describe and talk about autism in the current paper, given that this is the most preferred language of the autistic community [1, 2]. We recognise and respect the wide range of terms and different individual preferences for describing autism, and that the language used to describe and talk about autism will continue to evolve over time.
