# Supplementary material for: Development and validation of the suicidal behaviours questionnaire - autism spectrum conditions in a community sample of autistic, possibly autistic and non-autistic adults
Source: Mol Autism. 2021 Jun 21;12:46. doi: 10.1186/s13229-021-00449-3 (PMC8218414; doi:10.1186/s13229-021-00449-3)
Supplement: Supplementary file 1 — Additional file 1. Results of group comparisons between follow-up survey responders and non-responders. [file 13229_2021_449_MOESM1_ESM.docx]

Supplementary Information.

***Test Retest Reliability:*** N=172 autistic/possibly autistic participants completed the SBQ-ASC at time two out of the total n=421 participants who had completed the SBQ-ASC at time one (40.85%). N=72 non-autistic participants completed the SBQ-ASC at time two out of the n=268 participants who had completed the SBQ-ASC at time one (26.86%). Significantly more autistic/possibly autistic adults completed the SBQ-ASC at time one and time two (n=172) compared to non-autistic adults (n=72) (*X^2^*=14.26, *p*<.001). Independent samples *t-*tests and Pearson Chi-Square compared demographics between the sample who complete the SBQ-ASC at time one and time two, with those who only completed the SBQ-ASC at time one, in the autistic/possibly autistic group, and non-autistic group separately.

*Autistic/Possibly Autistic:* There were no significant differences in age (*t*(419)=.534, *p*=.064), sex ratio (*X^2^*=2.06, *p*<.151), rate of any developmental (*X^2^*=.15, *p*<.699) or mental health (*X^2^*=.453, *p*<.501) condition, or questionnaire scores (AQ (*t*(414)=.293, *p*=.078); CAT-Q (*t*(416)=.998, *p*=.71); INQ-10: Perceived Burdensomeness (*t*(404)=.503, *p*=.471) or Thwarted Belongingness (*t*(404)=.011, *p*=.987); ASA-A (*t*(413)=.479, *p*=.881); PHQ-9 (*t*(414)=.622, *p*=.914); SBQ-R (*t*(410)=.722, *p*=.197)), between the group of time two responders compared to non-responders.

*Non-Autistic:* There were no significant differences in age (*t*(266)=1.5, *p*=.124), sex ratio (*X^2^*=2.34, *p*<.126), rate of any developmental (*X^2^*=.0, *p*<.99) or mental health (*X^2^*=.07, *p*<.792) condition, or questionnaire scores (AQ (*t*(265)=1.34, *p*=.183); CAT-Q (*t*(266)=.858, *p*=.392); INQ-10: Perceived Burdensomeness (*t*(259)=1.26, *p*=.208) or Thwarted Belongingness (*t*(261)=.481, *p*=.449); ASA-A (*t*(265)=.201, *p*=.841); PHQ-9 (*t*(262)=1.27, *p*=.206); SBQ-R (*t*(263)=.216, *p*=.829)), between the group of time two responders compared to non-responders.
